# Supplementary material for: The efficacy of oxidized regenerated cellulose (SurgiGuard®) in breast cancer patients who undergo total mastectomy with node surgery: A prospective randomized study in 94 patients
Source: PLoS One. 2022 May 27;17(5):e0267694. doi: 10.1371/journal.pone.0267694 (PMC9140258; doi:10.1371/journal.pone.0267694)
Supplement: S1 File — (DOCX) [file pone.0267694.s002.docx]

| Research proposal |
| --- |
| **1. Title** |
| The efficacy of oxidized regenerated cellulose (SurgiGuard®) in breast cancer patients who undergo total mastectomy with node surgery: A prospective randomized study |
| **2. Investigator** |
| Principal Investigator: Yun Yeong Kim, MD, PhD  Faculty Advisor: Clinical Fellow Areum Jeong, Clinical Fellow Jeong-Won Na |
| **3. Faculty Director** |
| Yun Yeong Kim, MD, PhD  Department of General Surgery, Gachon University College of Medicine  38-13, Dokjeom-ro 3, Incheon, Republic of Korea |
| **4. Faculty Sponsor** |
| N/A |
| **5. Recruitment Institution** |
| Department of General Surgery, Gachon University College of Medicine  *Data approval requested to begin/end: IRB approval datae – 2020. 7.31* |
| **6. Target population** |
| Patients who will receive surgery with being diagnosed with breast cancer |
| **7. Materials** |
| Oxidized regenerated cellulose (ORC) is a topical hemostatic agent that has been in use for several decades. ORC acts hemostatically by absorbing blood, by surface interaction with platelets and proteins, and by activating the coagulation cascade. A novel ORC system, SurgiGuard® (Samyang Biopharmaceuticals Corp., Seoul, Korea), has been approved as a hemostatic agent by the Korean Food and Drug Administration (FDA). |
| **8. Purpose** |
| This study hypothesized that the hemostatic properties of this ORC would accelerate wound healing following breast and axillary surgery, including the removal of breast and axillary lymph nodes. ORC could reduce the accumulation of fluid resulting from transection of multiple small blood vessels and lymphatics, thereby reducing the duration and amount of serosanguinous drainage. The present study therefore compared the effects of SurgiGuard® plus closed suction drainage with those of suction drainage alone on seroma formation in patients undergoing total mastectomy and node surgery. High output and/or prolonged drainage tube use was regarded as an indication of increased risk of seroma formation. |
| **9. Sample size** |
| G*Power software (version 3.1.9.2) was used to determine the number of patients needed per group. A priori power calculations estimated that a minimum of 45 subjects in each arm would enable us to detect the difference with 80% power (alpha= 0.05) with a standard deviation of approximately 15%. We presumed drop rate would be around 10%. |
| **10. Inclusion and Exclusion criteria** |
| Inclusion criteria   1. Patients scheduled to undergo total mastectomy and node surgery (axillary lymph node dissection or sentinel lymph node biopsy) who were diagnosed with breast cancer. 2. Patients who understand the goal and the process of the trial and who want to participate in the trial with written informed consent. 3. No age limitation.   Exclusion criteria   1. Patients who had a personal history of hypersensitivity or allergic reaction to anticoagulants 2. Patients with obesity (defined as a body mass index >30 kg/m^2^) 3. Patients who received neoadjuvant chemotherapy or planned to undergo immediate breast reconstruction 4. Patients who have plan to palliative surgery |
| **11. Informed consent** |
| The study has been described to me and I understand that my participation is voluntary and that I am free to withdraw my consent and discontinue my participation in the project at any time without penalty. I also understand that the results of the study will be treated in strict confidence and reported as group data sets without personality identifying information, possibly in scholarly publications. I understand that if I have any questions or concerns about this experiment, that I may pose them to contact information here and Dr. Yun Yeong Kim (docsapphire@naver.com). |
| **12. Risk to participants** |
| Is there more than minimal risk of harm? N/A  What safeguards do you take to minimize those risks, if applicable? N/A |
| **13. Design and Procedure** |
| The patients were randomized into two groups, one treated with ORC plus closed suction drainage and the other with closed suction drainage alone. The randomization scheme utilized an allocation algorithm to ensure similar sample sizes at the end of patient accrual. Patients were randomized upon entering the operating room, at which time the surgeon opened the sealed envelope and read the group assignment card. Patients were blinded to their allocation throughout the course of the study.   \| **Schedule** \| **Visit 1**  (Day 0) \| **OP** \| **Visit** \| \| \| \| \| \| \| \| \| --- \| --- \| --- \| --- \| --- \| --- \| --- \| --- \| --- \| --- \| --- \| \| **Visit 2**  (POD#1) \| **Visit 3**  (POD#2) \| **Visit 4**  (POD#3) \| **Visit 5**  (POD#4) \| **Visit 6**  (POD#5) \| **Visit 7**  (POD#6) \| **Visit 8**  POD#7 ) \| **Visit 9**  POD#8 )  종료 \| \| *Informed consent* \| O \|  \|  \|  \|  \|  \|  \|  \|  \|  \| \| *P/Ex* \| O \|  \| O \| O \| O \| O \| O \| O \| O \| O \| \| *BW/Height* \| O \|  \|  \|  \| O \|  \| O \|  \|  \|  \| \| *Target drug* \|  \| O \|  \|  \|  \|  \|  \|  \|  \|  \| \| *Drain count (cc/day)* \|  \|  \| O \| O \| O \| O \| O \| O \| O \| O \| \| *Investigation/medicine* \| O \| O \| O \| O \| O \| O \| O \| O \| O \| O \| \| *Adverse effect* \|  \| O \| O \| O \| O \| O \| O \| O \| O \| O \| \| *Follow/up* \| POD#14, POD#21, loculated seroma amount by aspiration \| \| \| \| \| \| \| \| \| \| |
| **14. Standard Treatment of Participants** |
| Mastectomy and drain insertion followed by skin closure |
| **15. Benefits to participants and compensation** |
| This study stands in a position to greatly benefit this community. It will provide much needed information concerning the importance of seroma reduction, and the implication of applicant’s uses and possible outcome. Applied results will recommend ways patients might improve management and control of their quality of life. This information will be relayed via presentation and report to both the local people and greater scientific community. |
| **16. Data collection and outcomes** |
| Seroma amount check as drainage fluid amount  Electrocautery and ultrasonic dissection technology were used for hemostasis and lymphostasis. Wounds were irrigated with normal saline prior to wound closure, with excess liquid removed by drying with pads. Two separate suction drainage tubes were inserted, one into the breast and the other into axillary dead space. The total drainage volume was measured daily at the same time during hospitalization. The drainage tubes were removed when the amount of drainage was below 30 ml/day on at least two consecutive days. Compressive bandages were maintained by all patients until hospital discharge. |
| **17. Statistical analysis** |
| Categorical variables were compared by chi-square tests and continuous variables by Student’s t tests. All statistical analyses were performed IBM SPSS Statistics 19 software, with a *P* value <0.05 considered statistically significant. |
| **18. Subject Privacy and Data Confidentiality** |
| To help protect participants’ confidentiality, we will store these records in separate file and label them only by subject code. They will be seen only by the researcher analyzing their data, and will be destroyed in 3 years after data analysis is complete. For all other data, confidentiality will be maintained by using a numeric subject code in resulting data files. After the experiment, there will be no way to link their name with these data. When required by law, the records of this research may be reviewed by applicable government agencies. |
| **19. References** |
| 1. Agrawal A, Ayantunde AA, Cheung KL. Concepts of seroma formation and prevention in breast cancer surgery. ANZ J Surg. 2006;76:1088–1095.   2. Jain PK, Sowdi R, Anderson AD, MacFie J. Randomized clini-cal trial investigating the use of drains and fibrin sealant following surgery for breast cancer. Br J Surg. 2004;91:54–60.  3. Choi MS, Kim HK, Kim WS, Bae TH, Kim MK. A comparison of triamcinolone acetonide and fibrin glue for seroma prevention in a rat mastectomy model. Ann Plast Surg. 2012;69:209–212.  4. Kottayasamy Seenivasagam R, Gupta V, Singh G. Prevention of seroma formation after axillary dissection: A comparative randomized clinical trial of three methods. Breast J. 2013;19:478–484.  5. G. Qvamme, C.K. Axelsson, C.Lanng, M.Mortensen. Randomized clinical trial of prevention of seroma formation after mastectomy by local methylprednisolone injection. Br J Surg 2015;102:1195-203  6. Kim SH, Yoon HS, Kim HK. Efficacy of Oxidized Regenerated Cellulose, SurgiGuard, in Porcine Surgery. Yonsei Med J 2017; 58:195-205  7. Carlo Rassu. Observed outcomes on the use of oxidized and regenerated cellulose polymer for breast conserving surgery-A case series. Ann Med Surg (Lond) 2016 Feb; 5:57-66  8. G. Franceschini, G. Visconti, R. Masetti, Oncoplastic breast surgery with oxidized regenerated cellulose: appraisals based on five-year experience, Breast J. 20 (4) (2014) 447e448. |
