# Supplementary material for: The efficacy of oxidized regenerated cellulose (SurgiGuard®) in breast cancer patients who undergo total mastectomy with node surgery: A prospective randomized study in 94 patients
Source: PLoS One. 2022 May 27;17(5):e0267694. doi: 10.1371/journal.pone.0267694 (PMC9140258; doi:10.1371/journal.pone.0267694)
Supplement: S2 File — (DOCX) [file pone.0267694.s003.docx]

| 연구계획서 |
| --- |
| **1. 과제명과 단계** |
| 유방 전절제술후 장액종 형성 억제에 써지가드의 효과; 전향적 무작위 시험  The efficacy of oxidized regenerated cellulose (SurgiGuard®) in breast cancer patients who undergo total mastectomy with node surgery: A prospective randomized study |
| **2. 연구책임자, 공동연구자, 연구코디네이터, 관리약사의 성명과 직위** |
| 연구책임자: 조교수 김윤영  공동연구자: 임상강사 정아름, 임상강사 나정원 |
| **3. 연구의 의뢰자명과 주소** |
| 김윤영, 가천대학교 길병원 외과 유방암센터 |
| **4. 공동참여기관명·연구자 성명** |
| 해당없음 |
| **5. 연구의 수행장소 및 연구예상기간** |
| *연구장소: 가천대 길병원 외과*  *연구예상기간: IRB승인일 – 2020. 7.31* |
| **6. 연구대상질환** |
| 침윤성 유방암 및 유방 상피내암 진단 후 유방 전절제술 시행 예정인 환자 |
| **7. 연구의 배경 및 필요성** |
| 유방 전절제술 후 장액종 (seroma) 형성에 따른 처치법으로서 술후 일주일 이상의 도관거치는 필수적으로 행해져 왔다. 수술후 장액종 형성 억제를 위한 노력으로서 현재까지 알려진 방법으로는 hemostatic agent사용(SurgiGuard application), steroid application, physical dead space reduction등이 있고 이런 방법들로 장액종 형성 억제 시도후 도관 삽입없이 피부봉합을 시도해 볼 수 있다. 술후 장액종의 형성은 여러가지 요인의 복합적 결과로서 hemostasis시 조직에 가해지는 thermal injury, lymphatic and capillary disruption, inflammatory mediators 방출, dead space형성 등이 요인으로 여겨진다. SurgiGuard는 hemostatic agent로서 lymphatics sealing효과가 있고 dead space를 줄여주어 seroma 형성을 억제하는 역할을 한다. Surgiguard는 동물실험을 통해 지혈의 효과가 입증이 되어 2015년 시판이 시작되면서 주로 복부수술후 임상적으로 널리 쓰이고 있다. Surgiguard로써 유방 수술후 장액종 형성 억제에 대한 효과를 입증하는 연구는 아직까지는 없으나 breast conserving surgery시 Surgiguard의 사용으로 filling defect를 보완하는 필러로서의 Surgiguard의 효능에 대한 연구들이 있고 이들의 연구에서 기술하고 있는 Surgiguard의 합병증으로는 염증반응(농양, 피부염, 유선염 등)이 있다. 이들 연구에서 유방암을 떼어내고 유방모양을 형성해주는 parenchymal reshaping을 하면서 필러로서의 역할을 하게 되는 기전으로 Surgiguard가 fibrin deposition과 platelet aggregation으로 hemostasis를 돕고 3D구조를 형성하기 때문이라고 기술하고 있다. 임상적으로 seroma formation감소 효과가 어느정도 예측이 된 바, Surgiguard 제제가 얼마나 효과적이고 cost effective한지에 대해 전향적 연구를 진행하고자 한다. |
| **8. 연구의 목적과 가설** |
| Surgiguard가 술후 장액종 형성 억제에 얼마나 효과적인가를 확인하고자 함 |
| **9. 목표대상자 수와 산출근거 (Sample size)** |
| 본 연구 목적을 위해 요구되는 대상자의 수는 G*power 3.1 program으로 분석하였다. 분석에서 (t-test) 유의수준 0.05, 효과크기 0.7에서 95%의 power를 유지하기 위한 표본수는 그룹별로 총 45명이 필요했으나 탈락자를 예상(10%)하여 편의추출에 의해 총 100명의 대상자를 선정하였다.  (Translation as following)  G*Power software (version 3.1.9.2) was used to determine the number of patients needed per group. A priori power calculations estimated that a minimum of 45 subjects in each arm would enable us to detect the difference with 80% power (alpha= 0.05) with a standard deviation of approximately 15%. We presumed drop rate would be around 10%. |
| **10. 연구대상자의 선정기준/ 제외기준 (Inclusion and Exclusion criteria)** |
| *선정기준  1. 침윤성 유방암 진단 후 유방 전절제술 시행 예정인 환자  2. 본 연구에 대한 설명을 듣고 참여하기로 동의한 환자  3. 연령 제한은 없음  *제외기준  1. re-operation은 제외한다.  2. neoadjuvant Chemotherapy후의 수술과 palliative mastectomy는 제외하도록 한다.  3. Surgiguard 치료가 부적합한 자, 알러지나 감염이 있는 자  (Translation as following)  Inclusion criteria   1. Patients scheduled to undergo total mastectomy and node surgery (axillary lymph node dissection or sentinel lymph node biopsy) who were diagnosed with breast cancer. 2. Patients who understand the goal and the process of the trial and who want to participate in the trial with written informed consent. 3. No age limitation.   Exclusion criteria   1. Patients who had a personal history of hypersensitivity or allergic reaction to anticoagulants 2. Patients with obesity (defined as a body mass index >30 kg/m^2^) 3. Patients who received neoadjuvant chemotherapy or planned to undergo immediate breast reconstruction 4. Patients who have plan to palliative surgery |
| **11. 연구대상자 모집방법과 동의절차** |
| 상기 연구대상자의 선정기준을 만족하는 환자들을 대상으로 본 연구의 연구책임자 및 의뢰기관은 이 연구의 선정기준에 합당한 환자를 인종이나 사회경제적 상태를 이유로 부당하게 배제시키지 않을 것이다. 이 연구의 선정기준에 합당하다면 가능한 환자들이 이 연구에 참여할 수 있도록 모든 노력을 다할 것이며 본 기관에서 유방 전절제술을 받는 유방암 환자의 전체를 대표할 수 있도록 환자들에게 연구의 목적을 주지시킬 것이다. 연구책임자는 독립적인 장소에서 연구대상자가 연구에 대해 이해할 수 있도록 연구에 대해 충분히 설명하고 연구대상자가 자발적으로 참여할 수 있도록 노력할 것이다. |
| **12. 취약한 연구대상자를 포함하는 경우 추가적인 보호조치 방안** |
| 해당없음 |
| **13. 연구설계 및 연구방법(*Surgiguard는 제거하는 것이 아님)** |
| 1. 연구대상자에게 진행하게 될 연구절차에 대해 상세히 기술한다. 2. 검사항목(관찰항목)과 절차 및 방법: 하기 표에 기술된 관찰항목에 대해 방문시 측정한다. 3. 방문횟수: 재원기간 총 9회 4. 의약품의 용량과 투여 방법: 19번 항목에 적혀있음 5. 단일맹검법으로 연구진행; 환자에게 배정된 randomized group이 적힌 종이봉투는 mastectomy   종료전까지 개봉되지 아니하며, surgeon(공동연구자)이 피부봉합 전 개봉하여 종이에 적힌대로 시행후 피부봉합  연구가 끝나 데이터 분석이 끝날 때까지 맹검은 유지된다. Surgeon(공동연구자)은 환자가 어떤군에 속하는지에 대한 정보를 매 수술 종료시마다 하나의 파일에 모아둔다. 이는 암호화형태로 파일관리가 되며 연구가 끝나고 데이터 분석시 분석자에게 오픈된다.  (Translation as following, randomization protocol)  The patients were randomized into two groups, one treated with ORC plus closed suction drainage and the other with closed suction drainage alone. The randomization scheme utilized an allocation algorithm to ensure similar sample sizes at the end of patient accrual. Patients were randomized upon entering the operating room, at which time the surgeon opened the sealed envelope and read the group assignment card. Patients were blinded to their allocation throughout the course of the study.   1. 대조군은 현재 시행하고 있는 표준치료 방법으로서 mastectomy후 바로 피부봉합   대조군에 속한 환자들은 연구의 기대이익을 기대할 수 없지만 현재 행해지는 standard treatment를 받게 되므로 윤리적 문제는 극히 미미하다고 판단됨   1. 환자 각각이 어디에 배정되었는지는 수술 종료 후 연구 데이터 관리자(공동연구자)에게 전달되어 DB에 secured형태로 보관 2. 연구실행 일정표: 연구 착수에서부터 종료 시까지 연구대상자에게 시행되는 투약 및 각종 검사 등을 일시 별로 일목요연하게 정리한 일정표  \| **일정**  **관찰항목** \| **Visit 1**  (Day 0)  수술전 \| **OP** \| **방문 기간** \| \| \| \| \| \| \| \| \| --- \| --- \| --- \| --- \| --- \| --- \| --- \| --- \| --- \| --- \| --- \| \| **Visit 2**  (POD#1) \| **Visit 3**  (POD#2) \| **Visit 4**  (POD#3) \| **Visit 5**  (POD#4) \| **Visit 6**  (POD#5) \| **Visit 7**  (POD#6) \| **Visit 8**  POD#7 ) \| **Visit 9**  POD#8 )  종료 \| \| *동의서 취득* \| O \|  \|  \|  \|  \|  \|  \|  \|  \|  \| \| *활력상태/신체검사* \| O \|  \| O \| O \| O \| O \| O \| O \| O \| O \| \| *체중/신장* \| O \|  \|  \|  \| O \|  \| O \|  \|  \|  \| \| *임상약 투여* \|  \| O \|  \|  \|  \|  \|  \|  \|  \|  \| \| *Drain count (cc/day)* \|  \|  \| O \| O \| O \| O \| O \| O \| O \| O \| \| *복용 약물 조사* \| O \| O \| O \| O \| O \| O \| O \| O \| O \| O \| \| *이상반응 조사* \|  \| O \| O \| O \| O \| O \| O \| O \| O \| O \| \| *추적관찰* \| POD#14, POD#21에 loculation 되어 있는 seroma양 측정(aspiration을 통한 측정임) \| \| \| \| \| \| \| \| \| \| |
| **14. 대상질환의 표준치료 방법** |
| Mastectomy and drain insertion 후 skin closure |
| **15. 연구대상자의 위험과 이익** |
| 1. 연구참여로 인해 연구대상자에게 발생할 수 있는 위험이나 불편으로서 임상시험에 쓰이는 제제로 인한 합병증의 가능성이 있다. 수술부위 감염, 통증, 기타 예상치 못한 약제 부작용 2. 연구에 참여함으로써 어떤 시술 또는 처치, 행위가 예상치 못하는 위험을 수반할 수 있다는 사실 3. 연구에 참여함으로써 연구대상자에게 기대되는 이익   연구에서 예측되는 위험과 불편을 충분히 고려하여 연구대상자 개인과 사회가 얻을 수 있는 이익이 그 위험과 불편보다 크거나 이를 정당화할 수 있다고 판단되는 경우에만 연구의 타당성을 갖게 되며, 예측되는 불편이나 위험이 있는데도 연구를 수행해야 함은 사회적 이익 뿐 아니라 개인 차원에서도 위험의 가능성보다 이득의 가능성이 지극히 크기 때문이다. 연구에서 예측되는 개개인의 위험 발생 가능성은 상기약제의 국내 의약처 승인 내용에 따르면 상당히 미미할 것으로 생각된다. |
| **16. 유효성 평가항목 및 방법 (Data collection and outcomes)** |
| Seroma amount는 drainage fluid amount를 측정한다.  대조군에 비해 seroma의 양이 감소함을 측정하기 위한 방법으로   1. Seroma amount를 daily비교 (15%이상 감소시 유효함을 인정) 2. Drain removal의 기준은 seroma amount 30cc/day 이틀 이상 지속시로 하고 removal까지의 일수를 기록한다.   (Translation as following)  Electrocautery and ultrasonic dissection technology were used for hemostasis and lymphostasis. Wounds were irrigated with normal saline prior to wound closure, with excess liquid removed by drying with pads. Two separate suction drainage tubes were inserted, one into the breast and the other into axillary dead space. The total drainage volume was measured daily at the same time during hospitalization. The drainage tubes were removed when the amount of drainage was below 30 ml/day on at least two consecutive days. Compressive bandages were maintained by all patients until hospital discharge. |
| **17. 통계분석 방법(Statistical analysis)** |
| 연속변수로서의 seroma amount비교 (Student’s t tests)  (Translation as following)  Categorical variables were compared by chi-square tests and continuous variables by Student’s t tests. All statistical analyses were performed IBM SPSS Statistics 19 software, with a *P* value <0.05 considered statistically significant. |
| **18. 연구 중지·탈락 기준** |
| 1. 연구대상자가 참여동의를 철회한 경우 2. 중대한 이상반응이나 갑작스러운 사고로 인하여 연구를 계속할 수 없는 경우 3. 계획서에 명시된 방문 및 절차를 준수하지 않으려 하거나 할 수 없을 때 4. 중증의 합병증의 발현으로 연구 지속이 곤란한 경우 5. 선정기준에 적합하지 않은 환자가 연구에 참여한 경우 6. 기타 연구를 지속하는데 지장을 준다고 연구책임자가 판단한 경우) |
| **19. 연구에 사용되는 의약품 (약제정보는 제품설명서 첨부 참조)** |
| 1. 써지가드   한미약품, SugiGuard 써지가드 거즈 10cmx10cm(1장)  Absorbable hemostat, Oxidized regenerated cellulose  약제는 피부 봉합 직전에 1회 도포한다. 이는 제거되지 않으며 조직에 남아 반응을 일으킨다. 술후 추가적인 투여는 없으며 drain tube를 통해 나오는 seroma 측정을 통해 약물효과를 관찰하게 된다.  예상되는 부작용으로 상처회복 지연 및 피부괴사, 혈관협착, 감염 등이 있다. |
| **20. 예측 부작용과 사용상의 주의사항** |
| 상처회복 지연 및 피부괴사, 혈관협착, 감염 가능성이 있음 |
| **21. 부작용을 포함한 안전성의 평가기준, 평가방법과 보고방법** |
| 연구책임자는 본 계획서에 명시된 이상반응 또는 중대한 이상반응의 정의와 기준에 해당되는 증상들을 발견하고 문서화할 책임이 있다. 연구책임자는 연구기간 동안 연구대상자에게 적절한 의학적 치료를 할 책임이 있다. 연구책임자는 적절한 치료를 통하여 중대하거나 연구종료 전에 연구대상자를 중단시킨 원인이 된 이상반응을 추적관찰 할 책임이 있다. 연구대상자는 그 반응이 소실되거나 설명될 때까지 추적관찰하며 추적 방문의 횟수는 연구책임자의 판단에 따른다.  이상반응의 정의  이상반응이란 환자 또는 연구대상자에게 시험약 사용과 일시적으로 연관된 바람직하지 않은 모든 의학적 상태로서, 반드시 시험물질과 인과관계를 가져야 하는 것은 아니다.  중대한 이상반응(Serious Adverse Events)의 정의  시험약을 사용함에 있어 아래에 해당하는 경우를 말한다.  (1) 사망을 초래하거나 생명을 위협하는 경우  (2) 입원 또는 입원기간의 연장이 필요한 경우  (3) 지속적 또는 의미 있는 불구나 기능저하를 초래하는 경우  (4) 선천적 기형 또는 이상을 초래하는 경우  (5) 기타 연구책임자가 의학적으로 중증질환이라고 판단한 경우  이상반응 및 중대한 이상반응 기록  연구책임자는 증례기록서에 이상반응 및 중대한 이상반응과 관련된 모든 정보 즉, 이상 반응명, 발생일, 종료일, 강도, 시험물질과의 연관성, 결과, 치료여부, 중대한 이상반응 여부에 대한 기록을 하며, IRB에 이를 알린다.  이상반응 및 중대한 이상반응의 처치  이상반응을 경험한 모든 환자는 증상이 소실되고 비정상적 임상 검사치가 정상치로 회복되거나, 혹은 관찰된 변화에 대해 만족스러운 설명이 있을 때까지 가능한 모니터링 해야 한다. 또한 이상반응에 관한 모든 관찰 결과는 증례기록서와 환자 의무기록지에 기록한다.  중대한 이상반응의 보고  연구책임자가 전혀 예측하지 못하는 매우 드문 부작용이 발생할 경우 연구책임자 또는 담당자는 중대한 이상반응을 연구책임자가 알게 된 시점에서 7일 이내에 중대한 이상반응 보고양식을 이용하여 가천대 길병원 임상연구윤리심의위원회(IRB)에 보고하며, 식품의약품안전처장에게 보고한다. 필요한 경우 연구책임자 또는 담당자는 중대한 이상반응에 대한 새로운 정보를 포함하는 추적조사 보고서를 작성하여 가천대 길병원 임상연구윤리심의위원회 및 식품의약품안전처장에게 송부한다.) |
| **22. 연구대상자의 보상방안** |
| 1. 임상시험의 참여로 인한 대상자의 신체적 손상이 발생할 경우, 금전적 보상에 대하여 확정되기 전이라도 임상시험책임자(담당자)를 통한 적절한 치료 또는 치료 기회를 우선적으로 제공한다. 2. 임상시험용의약품에 의해 발생한 이상반응이나 이상반응 처치 과정에서 발생된 손상이 있는 경우도 보상 대상으로 고려한다. 3. 해당 이상반응으로 인한 손상이 예상되었으며 임상시험 대상자가 자발적으로 해당 임상시험 참여에 동의하였다 하더라도 보상 대상으로 고려한다. 4. 연구에 참여함으로써 대상자가 부담해야 하는 비용은 없다. (연구비에서 조달예정, 제약회사측에서 자체 프로세스를 통해 낮은 가격으로 약품 제공 예정임) |
| **23. 개인정보 보호 및 연구자료의 기밀 유지를 위한 방안** |
| 연구 진행을 위해 연구대상자의 개인정보(성명, 병록번호 등)와 민감정보(의학적 과거력, 병력, 유전정보 등)가 수집되지만 신원을 파악할 수 있는 기록은 관리번호를 부여하여 익명화하고 패스워드가 걸린 파일로 컴퓨터에 저장되고, 연구관련 자료는 잠금 장치가 있는 연구실에 보관하도록 하여 엄격하게 비밀로 유지되고 보호를 받으며, 본 연구의 모든 결과는 병원 파일에 기록되며 의뢰자 및 식품의약품안전처에서 치료의 상태 및 유효성을 평가하기 위해 검토하게 된다. 모니터요원, 점검을 실시하는 자, 심사위원회 및 식품의약품안전처장은 연구대상자의 비밀보장을 침해하지 않고 관련 규정이 정하는 범위 안에서 연구의 실시절차와 자료의 신뢰성을 검증하기 위해 연구대상자의 의무기록을 직접 열람할 수 있다. 연구대상자의 신원을 파악할 수 있는 기록은 비밀로 보장될 것이며 연구의 결과가 출판될 경우에도 연구대상자의 자료는 익명으로 처리되어 보호를 받게 된다. 연구대상자는 개인정보 수집 및 이용에 대한 동의를 거부할 수 있는 권리가 있으며, 동의를 거부한 경우 본 연구에 참여하지 않는다. 본 연구를 위해 수집한 개인정보와 연구 관련 모든 자료는 연구종료 후 3년간 보관될 것이며 다른 연구에 제공하지 않고 본 연구에만 사용하며, 보관기간이 지난 후 파기할 것이다.) |
| **24. 중간분석에 대한 계획 및 필요 시 연구의 조기 종료하는 범위를 포함하여 연구의 통계 분석에 대한 계획 기술** |
| 해당없음 |
| **25. 자료안전모니터링 계획(DSMP)** |
| 연구책임자는 안전성 정보(AE, SAE)을 모니터링 할 것이고 증례기록지 작성 시에 주기적으로 검토가 이루어질 것이며, 이상반응 발생 시 IRB에 보고할 것이다. 연구책임자는 임상시험 계획서의 내용에 따라 임상시험의 지속 혹은 중단을 결정할 것이다.  이 임상시험에 등록된 대상자의 지속적인 안전을 보증하기 위해 지속적으로 데이터를 모니터하고 담당자에게 알릴 것이다. |
| **26. 참고문헌** |
| 1. Agrawal A, Ayantunde AA, Cheung KL. Concepts of seroma formation and prevention in breast cancer surgery. ANZ J Surg. 2006;76:1088–1095.   2. Jain PK, Sowdi R, Anderson AD, MacFie J. Randomized clini-cal trial investigating the use of drains and fibrin sealant following surgery for breast cancer. Br J Surg. 2004;91:54–60.  3. Choi MS, Kim HK, Kim WS, Bae TH, Kim MK. A comparison of triamcinolone acetonide and fibrin glue for seroma prevention in a rat mastectomy model. Ann Plast Surg. 2012;69:209–212.  4. Kottayasamy Seenivasagam R, Gupta V, Singh G. Prevention of seroma formation after axillary dissection: A comparative randomized clinical trial of three methods. Breast J. 2013;19:478–484.  5. G. Qvamme, C.K. Axelsson, C.Lanng, M.Mortensen. Randomized clinical trial of prevention of seroma formation after mastectomy by local methylprednisolone injection. Br J Surg 2015;102:1195-203  6. Kim SH, Yoon HS, Kim HK. Efficacy of Oxidized Regenerated Cellulose, SurgiGuard, in Porcine Surgery. Yonsei Med J 2017; 58:195-205  7. Carlo Rassu. Observed outcomes on the use of oxidized and regenerated cellulose polymer for breast conserving surgery-A case series. Ann Med Surg (Lond) 2016 Feb; 5:57-66  8. G. Franceschini, G. Visconti, R. Masetti, Oncoplastic breast surgery with oxidized regenerated cellulose: appraisals based on five-year experience, Breast J. 20 (4) (2014) 447e448. |
