# Supplementary material for: The efficacy of oxidized regenerated cellulose (SurgiGuard®) in breast cancer patients who undergo total mastectomy with node surgery: A prospective randomized study in 94 patients
Source: PLoS One. 2022 May 27;17(5):e0267694. doi: 10.1371/journal.pone.0267694 (PMC9140258; doi:10.1371/journal.pone.0267694)
Supplement: Attachment — Submitted filename: review1.docx [file pone.0267694.s004.docx]

**Manuscript Number:** PONE-D-20-33439

**Manuscript Title:** The efficacy of oxidized regenerated cellulose (SurgiGuard®) in breast cancer patients who undergo total mastectomy with node surgery: A prospective randomized study in 94 patients

This study is a single blinded, prospective randomized controlled trial conducted at a single specialized breast cancer center in order to value the efficacy of oxidized regenerated cellulose (ORC) in breast cancer patients who undergo total mastectomy with node surgery.

This study was registered at Clinical Research Information Service, Republic of Korea (KCT0005637).

Ninetyfour breast cancer patients were enrolled in this study. The patients were randomized into two groups, one treated with ORC plus closed suction drainage and the other with closed suction drainage alone.

After an analysis of the results, the Authors conclude that use of ORC did not significantly alter the risk of seroma formation.

However, the manuscript falls under the scope of journal; It is an interesting topic. The study presents the results of original research.

The article is presented in an intelligible fashion; The work is described expansively; it is well structured and developed.

The manuscript is written in standard English; Language and grammar are acceptable. There are some typos.

The abstract is appropriate with the study; The introduction is adequate and clear. Materials and Methods are clear and linear.

Experiments, statistics, and analyses are performed to a technical standard and are described in sufficient detail; Categorical variables were compared by chi-square tests and continuous variables by Student’s t tests. All statistical analyses were performed IBM SPSS statistics 19 software, with a p value <0.05 considered statistically significant.

Results reported have not been published elsewhere.

The discussion and conclusions should be improved:

- Discussion: the Authors should describe better the benefits and issues due to the use of ORC in breast surgery (see new references); description of histologic features resulting from the interaction between tissues and ORC may be very important in understanding the physiologic processes behind ORC integration and local side effects.
- The conclusions are supported by the data but should be better explained and presented in an appropriate fashion.

Some specific references about use of ORC in breast surgery should be read and added in the discussion:

- Franceschini G, Visconti G, Sanchez AM, Di Leone A, Salgarello M, Masetti R. Oxidized regenerated cellulose in breast surgery: experimental model. J Surg Res. 2015 Sep;198(1):237-44. doi: 10.1016/j.jss.2015.05.012.
- Franceschini G. Internal surgical use of biodegradable carbohydrate polymers. Warning for a conscious and proper use of oxidized regenerated cellulose. Carbohydr Polym. 2019 Jul 15;216:213-216. doi: 10.1016/j.carbpol.2019.04.036.
- Rassu PC. Observed outcomes on the use of oxidized and regenerated cellulose polymer for breast conserving surgery - A case series. Ann Med Surg (Lond). 2015 Dec 22;5:57-66. doi: 10.1016/j.amsu.2015.12.050.
- Rassu PC, Serventi A, Giaminardi E, Ferrero I, Tava P. Use of oxidized and regenerated cellulose polymer in oncoplastic breast surgery. Ann Ital Chir. 2013 Jan 29;84(ePub):S2239253X13020288.
- Franceschini G, Visconti G, Terribile D, Fabbri C, Magno S, Di Leone A, Salgarello M, Masetti R. The role of oxidized regenerate cellulose to prevent cosmetic defects in oncoplastic breast surgery. Eur Rev Med Pharmacol Sci. 2012 Jul;16(7):966-71.
- Franceschini G, Sanchez AM, Visconti G, Di Leone A, Salgarello M, Masetti R. Quadrantectomy with oxidized regenerated cellulose ("QUORC"): an innovative oncoplastic technique in breast conserving surgery. Ann Ital Chir. 2015;86:548-52.

**CONSORT checklist:**

**Outcomes (Item 6a): The Authors should better describe outcomes and completely defined pre-specified primary outcome measure including how and when it was assessed.**

**Sample size (Item 7a): it was determined by**
G*Power software (version 3.1.9.2). A priori power calculations estimated that a minimum of 45 subjects in each arm would enable to detect the difference with 80% power (alpha= 0.05) with a standard deviation of approximately 15%. The Authors presumed drop rate would be around 10%.

**Sequence generation (Item 8a), Allocation concealment (Item 9), Blinding (Item 11a):** The randomization scheme utilized an allocation algorithm to ensure similar sample sizes at the end of patient accrual. Patients were randomized upon entering the operating room, at which time the surgeon opened the sealed envelope and read the group assignment card. Patients were blinded to their allocation throughout the course of the study.

However, the type of allocation algorithm should be better described; are outcome assessors blinded?
**Outcomes and estimation (Item 17a/b): they should be revised.
Harms (Items 19): All important harms and unintended effects in each group** are reported.
**Registration (Item 23):** This study was registered at Clinical Research Information Service (CRiS), Republic of Korea (KCT0005637).

**Protocol (Item 24):** The study protocol was approved by the Institutional Review Board of Gil hospital (IRB No. GCIRB 2019-150).

**Funding (Item 25):** This work was supported by Gachon University research fund of 2017 (GCU-2017- 5258). However, the **role of funders**
should be better described.

**The manuscript may be accepted with some revisions.**
