# Supplementary material for: The efficacy of oxidized regenerated cellulose (SurgiGuard®) in breast cancer patients who undergo total mastectomy with node surgery: A prospective randomized study in 94 patients
Source: PLoS One. 2022 May 27;17(5):e0267694. doi: 10.1371/journal.pone.0267694 (PMC9140258; doi:10.1371/journal.pone.0267694)
Supplement: Attachment — Submitted filename: Response to Reviewers.docx [file pone.0267694.s005.docx]

**Reviewer #1: Manuscript Number: PONE-D-20-33439**

Manuscript Title: The efficacy of oxidized regenerated cellulose (SurgiGuard®) in breast cancer patients who undergo total mastectomy with node surgery: A prospective randomized study in 94 patients

This study is a single blinded, prospective randomized controlled trial conducted at a single specialized breast cancer center in order to value the efficacy of oxidized regenerated cellulose (ORC) in breast cancer patients who undergo total mastectomy with node surgery.
This study was registered at Clinical Research Information Service, Republic of Korea (KCT0005637).
Ninety four breast cancer patients were enrolled in this study. The patients were randomized into two groups, one treated with ORC plus closed suction drainage and the other with closed suction drainage alone.
After an analysis of the results, the Authors conclude that use of ORC did not significantly alter the risk of seroma formation.

The manuscript falls under the scope of journal; It is an interesting topic. The study presents the results of original research.
The article is presented in an intelligible fashion; The work is described expansively; it is well structured and developed.
The manuscript is written in standard English; Language and grammar are acceptable. There are some typos.
The abstract is appropriate with the study; The introduction is adequate and clear. Materials and Methods are clear and linear.
Experiments, statistics, and analyses are performed to a technical standard and are described in sufficient detail; Categorical variables were compared by chi-square tests and continuous variables by Student’s t tests. All statistical analyses were performed IBM SPSS statistics 19 software, with a p value <0.05 considered statistically significant.
Results reported have not been published elsewhere.

1. The discussion and conclusions should be improved:
- Discussion: the Authors should describe better the benefits and issues due to the use of ORC in breast surgery (see new references); description of histologic features resulting from the interaction between tissues and ORC may be very important in understanding the physiologic processes behind ORC integration and local side effects.
- The conclusions are supported by the data but should be better explained and presented in an appropriate fashion.

Some specific references about use of ORC in breast surgery should be read and added in the discussion:
- Franceschini G, Visconti G, Sanchez AM, Di Leone A, Salgarello M, Masetti R. Oxidized regenerated cellulose in breast surgery: experimental model. J Surg Res. 2015 Sep;198(1):237-44. doi: 10.1016/j.jss.2015.05.012.
- Franceschini G. Internal surgical use of biodegradable carbohydrate polymers. Warning for a conscious and proper use of oxidized regenerated cellulose. Carbohydr Polym. 2019 Jul 15;216:213-216. doi: 10.1016/j.carbpol.2019.04.036.
- Rassu PC. Observed outcomes on the use of oxidized and regenerated cellulose polymer for breast conserving surgery - A case series. Ann Med Surg (Lond). 2015 Dec 22;5:57-66. doi: 10.1016/j.amsu.2015.12.050.
- Rassu PC, Serventi A, Giaminardi E, Ferrero I, Tava P. Use of oxidized and regenerated cellulose polymer in oncoplastic breast surgery. Ann Ital Chir. 2013 Jan 29;84(ePub):S2239253X13020288.
- Franceschini G, Visconti G, Terribile D, Fabbri C, Magno S, Di Leone A, Salgarello M, Masetti R. The role of oxidized regenerate cellulose to prevent cosmetic defects in oncoplastic breast surgery. Eur Rev Med Pharmacol Sci. 2012 Jul;16(7):966-71.
- Franceschini G, Sanchez AM, Visconti G, Di Leone A, Salgarello M, Masetti R. Quadrantectomy with oxidized regenerated cellulose ("QUORC"): an innovative oncoplastic technique in breast conserving surgery. Ann Ital Chir. 2015;86:548-52.

***🡺Thank you for these kind comments and for the recommendations. We tried our best to reflect your recommendations. We added a paragraph explaining benefits and integration issues of ORC in breast cancer surgery by references that you provided.***

2. CONSORT checklist:

Outcomes (Item 6a): The Authors should better describe outcomes and completely defined pre-specified primary outcome measure including how and when it was assessed.
Sample size (Item 7a): it was determined by
G*Power software (version 3.1.9.2). A priori power calculations estimated that a minimum of 45 subjects in each arm would enable to detect the difference with 80% power (alpha= 0.05) with a standard deviation of approximately 15%. The Authors presumed drop rate would be around 10%.

Sequence generation (Item 8a), Allocation concealment (Item 9), Blinding (Item 11a): The randomization scheme utilized an allocation algorithm to ensure similar sample sizes at the end of patient accrual. Patients were randomized upon entering the operating room, at which time the surgeon opened the sealed envelope and read the group assignment card. Patients were blinded to their allocation throughout the course of the study.
However, the type of allocation algorithm should be better described; are outcome assessors blinded?
Outcomes and estimation (Item 17a/b): they should be revised.
Harms (Items 19): All important harms and unintended effects in each group are reported.

Registration (Item 23): This study was registered at Clinical Research Information Service (CRiS), Republic of Korea (KCT0005637).

Protocol (Item 24): The study protocol was approved by the Institutional Review Board of Gil hospital (IRB No. GCIRB 2019-150).

***🡺Thank you for the valuable comments. We found that some shortcomings were observed and improved the manuscript in method section according to CONSORT 2010 guideline.***

3. Funding (Item 25):

This work was supported by Gachon University research fund of 2017 (GCU-2017- 5258). However, the role of funders should be better described.

***🡺Thank you for the comment. This work was supported by Gachon University research fund and there were no conflicts. The budget was directly attributed to purchase of the material(******SurgiGuard®). We added this description to the literature.***

The manuscript may be accepted with some revisions.

**Reviewer #2: The objective of this prospective, randomized controlled trial (RCT) is to assess the effectiveness of the "ORC + closed suction drainage", over the "only closed suction drainage" procedures in breast cancer patients who underwent total mastectomy.**

The study was registered as a RCT within the Korean Clinical Trial Registry (with a legit number), and was approved by the respective IRB/Ethics Committee. While the study objectives sound interesting, is important, and on target, some shortcomings were observed, in regards to abiding by the CONSORT guidelines for conducting and reporting results of high-quality randomized controlled trials (RCTs). Some other (statistical) comments were also provided.


1. Methods:

Methods reporting appeared very messy. An orderly manner is suggested, following CONSORT guidelines, without repeating information, such as Trial Design, Participant Eligibility Crtieria and settings, Interventions, Outcomes, sample size/power considerations, Interim analysis and stopping rules, Randomization (details on random number generation, allocation concealment, implementation), Blinding issues, etc, should be mentioned.

The authors are advised to create separate subsections for each of the possible topics (whichever necessary), and that way produce a very clear writeup. I see the Authors indeed made an attempt; a nice example can be seen in the manuscript below:

<https://www.sciencedirect.com/science/article/pii/S0889540619300010>

***🡺Thank you for the valuable comments. We found that some shortcomings were observed and improved the manuscript in method section according to CONSORT 2010 guideline.***

Specific comments below:

(a) For instance, the randomization and allocation concealment should be made very clear (they are NOT the same thing); the trial staff recruiting patients should NOT have the randomization list. Randomization should be prepared by the trial statistician, and he/she would not participate in the recruiting.
(b) More details on the randomization is necessary. For example, was it a block randomization?. If Yes, then what's the block size?

***🡺 Thank you for your specific kind comments. We improved method section as your opinion.***

(c) Sample size/power: The sample size/power computations should be conducted using the primary response; it is not clear what the authors did. Furthermore, the calculations should clearly state the "effect size" under consideration. Taking a standard deviation of ~ 15% doesn't convey much information.

**🡺Thank you for the sharp observation. We added “effect size” statement with Table 3 according to your recommendation. We calculated binary outcomes for duration of hospitalization based on POD 10 and described in the result section.**

(d) Statistical Analysis: Overall, looks OK, and straightforward. Standard 2-sample t-tests were used. Did the authors confirm that Gaussian assumptions were valid? Otherwise, they would have resorted to nonparametric tests, such as various Wilcoxon tests.

***🡺 Thank you for your valuable comments. We analyzed and just compared continuous variable(mean amount of seroma) in both groups. We did not intend to compare the tendency of seroma amount variation in relation to time elapse. Therefore, we did not confirm the Gaussian assumptions or Wilcoxon test.***

2. Results & Conclusions:

(a) The authors should check that any statement of significance should be followed by a p-value in the entire Results section.

***🡺Thank you for your sharp comments. We stated P value significance in the statistical analysis section and checked for entire results section. We described our null findings in the study as “similar in these two groups” in the literature and added a paragraph as your opinion with statement of “no statistically difference between the two groups (Table 3)”.***

(b) Null findings were observed (no differences between the groups). The Conclusions section should clearly state that the results/conclusions are "only" from this Korean population, and allude to future studies, sometimes multicenter, with possibly higher sample sizes, and/or combining other populations to evaluate the difference further.

***🡺Thank you for your kind comments. We added limit of our study in that the outcomes depend only on Korean populations in last paragraph of discussion.***

**Reviewer #3: Dear Author,**

This is a well written paper on a subject that is highly relevant for breast cancer patients undergoing mastectomy. Seroma formation results in the risk of SSI, more visits and interventions, higher costs, decreased satisfaction and potential delay in adjuvant treatment and/or return to work. Several interventions have been investigated over the years, but results are not very satisfying. Although the methods are well described there are some major flaws in the methods chosen and some important data/analysis is lacking. If the below comments are addressed well I would think this paper is relevant and should definitely be published. Especially because wound care intervention studies are scarce, especially RCTs. It is important to also publish negative studies, as future studies could repeat this intervention with improved methods potentially finding a benefit of this theoretical smart and patient friendly intervention.

***🡺Thank you for these kind comments and for the recommendations. We tried our best to reflect your recommendations. And we prepared following point by point response.***

Introduction
Please add a paragraph on the relevance of seroma formation for patients and healthcare. More and longer seroma duration has the risk of SSI, more visits and interventions, higher costs, decreased satisfaction and potential delay in adjuvant treatment and/or return to work.

***🡺Thank you for the valuable comment. We added a sentence for seroma healthcare issue in first paragraph of introduction and added SSI issues in the discussion section as you recommended.***

Methods
1. The intervention itself is not clearly described. In the current manuscript just very briefly the SurgiGuard product working mechanism is described in the introduction, but the exact application is unclear for a reader. please ellaborate more on this, as this is crucial for the potential user group. I assume the product is left in the wound bed before closing the mastectomy wound. Please also describe in detail the surgical procedure. Wat settings of electrocautery were used? What kind of suture technique?

***🡺Thank you for the valuable comments. We found that some shortcomings were observed and improved the manuscript in method section according to CONSORT 2010 guideline. In the intervention section, we added additional description as your opinion.***

2. exclusion criteria: why is a BMI >30 used as an exclusion. It is a known risk factor for wound infection and seroma formation.

***🡺Yes, we agree with your opinion. We added a sentence to description of exclusion criteria.***

3. Wound complications: this subject should be discussed in more detail. What specific wound complications: infection (according to which criteria, preferrably CDC criteria), dehiscense, necrosis. This is very relevant as infections are the major cause of morbidity and costs in this patient group. Also the occurence of seroma/hematoma is highly correlated to the occurence of wound infection. (refs El-Tamer 2007, Xue 2012, Mukesh 2012 and Struik 2018)

***🡺We added SSI issues in the discussion section and improved the manuscript. Thank you for your nice recommendation.***

4. I would be interested in postoperative Hemoglobine decline and did you collect data on blood loss during surgery and compare this between groups?

***🡺Thank you for your interest. We think Hb decline was due to postoperative fluid loading rather than due to significant bleeding during surgery. We did not collect blood loss amount data during surgery.***

Results
1. There is a strikingly long mean drainage time in your study, compared to other studies. Long drainage time is a known risk factor for infection and should therefore be avoided. Also this results in very long mean hospital stays**.** Please ellaborate on this more and compare to literature. The long drainage time is probably impacting on your results. DId you consider to use other endpoints such as CDC criteria for seroma, number of reintervention and f.e. US measurements of seroma volumes instead?

***🡺Thank you for your sharp observation. Participants were given for informed consent for longer hospitalization and examined thoroughly under daily inspection. The higher mean total seroma volume observed in our patients may have been due to longer duration of hospitalization with drainage tubes. We added a paragraph about SSI issue in relation to longer drainage time in the literature. We demonstrated and compared seroma amount and post-discharge # of aspiration of wound in both groups. Those results were in table 2.***

Discussion
please compare especially drainage time, SSI and potential use of other seroma related outcomes to literature.

***🡺We added SSI issues in the discussion section and improved the manuscript. Thank you for your nice recommendation.***
