# Supplementary material for: The efficacy of oxidized regenerated cellulose (SurgiGuard®) in breast cancer patients who undergo total mastectomy with node surgery: A prospective randomized study in 94 patients
Source: PLoS One. 2022 May 27;17(5):e0267694. doi: 10.1371/journal.pone.0267694 (PMC9140258; doi:10.1371/journal.pone.0267694)
Supplement: Attachment — Submitted filename: COMMENTS TO THE AUTHORS.docx [file pone.0267694.s006.docx]

**COMMENTS TO THE AUTHORS**

This is a very good initiative and it will be an initial step in determining the role of ORC in seroma prevention after mastectomy. Studies like this will go a long way in reducing the ever-present problem of seroma after mastectomy but there some clarifications we need from the author before publication

1. The authors registered the clinical trial on 25^th^ November, 2020 while the patient enrollment started from 01^st^ June 2019 to 31^st^ August 2020. The registration was done 4 months after completion of the study which is against the ICMJE’s clinical trial registration policy. There is need for the clarification from the authors.
2. It is evident that the authors have a good grasp of seroma, but as part of the introduction, there is need to briefly mention proposed theories for development of seroma after mastectomy. Also, there is need to briefly mention the effect of seroma on patients, surgical team and the hospital community.
3. The authors mention high drain output and prolonged drainage as risk for seroma development after mastectomy. However, there is an oversight as the reference for this wasn’t cited.
4. The authors description of data collection protocol should be detailed and should include detailed preoperative data collection protocol, detailed intraoperative protocol including the protocol for placement of ORC in the patients, and detailed post operative protocol including the protocol for diagnosing seroma.
5. The authors also need to be detailed in the description of the study design. Is it a superiority design or a non-inferiority design?
6. There is need for the authors to highlight the incidence of seroma in each group and the overall incidence of seroma in the study.
7. Wound complications developed by some patients should be specified and the measures taken to handle them should be highlighted.
8. The authors gave a detailed discussion but there is need to organize it better to improve flow and readability. Also, there is no need to discuss lymphedema as part of the manuscript.
