# Supplementary material for: The efficacy of oxidized regenerated cellulose (SurgiGuard®) in breast cancer patients who undergo total mastectomy with node surgery: A prospective randomized study in 94 patients
Source: PLoS One. 2022 May 27;17(5):e0267694. doi: 10.1371/journal.pone.0267694 (PMC9140258; doi:10.1371/journal.pone.0267694)
Supplement: Attachment — Submitted filename: Response to Reviewers.docx [file pone.0267694.s007.docx]

**COMMENTS TO THE AUTHORS**

This is a very good initiative and it will be an initial step in determining the role of ORC in seroma prevention after mastectomy. Studies like this will go a long way in reducing the ever-present problem of seroma after mastectomy but there some clarifications we need from the author before publication

1. The authors registered the clinical trial on 25^th^ November, 2020 while the patient enrollment started from 01^st^ June 2019 to 31^st^ August 2020. The registration was done 4 months after completion of the study which is against the ICMJE’s clinical trial registration policy. There is need for the clarification from the authors.

We agree with your point. This study is prospective designed study but there were some errors in our study team, therefore we were late for registration to public repository system.

We describe this explanation adding one sentence in the method section.

2. It is evident that the authors have a good grasp of seroma, but as part of the introduction, there is need to briefly mention proposed theories for development of seroma after mastectomy. Also, there is need to briefly mention the effect of seroma on patients, surgical team and the hospital community.

According to your points, we added several sentences with brief mention to previous 1^st^ and second paragraph of introduction section. Thank you.

3. The authors mention high drain output and prolonged drainage as risk for seroma development after mastectomy. However, there is an oversight as the reference for this wasn’t cited.

According to your points, we added several sentences with brief mention to previous 1^st^ paragraph of introduction section. Thank you.

4. The authors description of data collection protocol should be detailed and should include detailed preoperative data collection protocol, detailed intraoperative protocol including the protocol for placement of ORC in the patients, and detailed post operative protocol including the protocol for diagnosing seroma.

Thank you for your detailed comments, and we added some descriptions to the protocol. Our original manuscript included details for readers to make sense the whole study process, but we agree with your point. We found that post-discharge process details were not cited, and added the details in the postoperative data collection.

5. The authors also need to be detailed in the description of the study design. Is it a superiority design or a non-inferiority design?

Yes, we added the comment in the study design description. Superiority design with ORC efficacy, of course. Thank you for your sharp point.

6. There is need for the authors to highlight the incidence of seroma in each group and the overall incidence of seroma in the study.

As you know, postoperative seroma incidence is one hundred percent. This study is about the efficacy of ORC reducing the seroma formation.

7. Wound complications developed by some patients should be specified and the measures taken to handle them should be highlighted.

Thank you for your sharp point. We added the description to the discussion section.

8. The authors gave a detailed discussion but there is need to organize it better to improve flow and readability. Also, there is no need to discuss lymphedema as part of the manuscript.

This study is about the efficacy of ORC use. We compared the outcome of each group, and did not find the superiority of the ORC for reducing the seroma formation.

In mastectomy with axillary surgery, the damage of lymphatic passage entails the massive fluid collection. Discussion about lymphedema seems to be out of subject, but we think that it is worthy to be highlighted in the view of ORC efficacy. Thank you for your detailed review and it was helpful for the completeness of the manuscript.
