# Supplementary material for: The efficacy of oxidized regenerated cellulose (SurgiGuard®) in breast cancer patients who undergo total mastectomy with node surgery: A prospective randomized study in 94 patients
Source: PLoS One. 2022 May 27;17(5):e0267694. doi: 10.1371/journal.pone.0267694 (PMC9140258; doi:10.1371/journal.pone.0267694)
Supplement: Attachment — Submitted filename: Response to Reviewers.docx [file pone.0267694.s008.docx]

**COMMENTS TO THE AUTHORS**

Dear authors,
You have addressed most of my concerns as requested. However, in the intervention, you may need to mention how the mastectomy was performed. You don't need to describe the procedure, just refer your readers to a manuscript that describe the procedure and cite it in you article. Also in your discussion, it will be good if you compare your findings to other studies in Africa, Europe and Americas.
Thank you.

Response:

Dear Editors,

Thank you for your kind comments. According to your recommendations, we added a mention about mastectomy in reference to conventional procedure website. Plus, we included other two similar

comparison studies documenting especially about ‘seroma formation’, those are in few of ORC-related

randomized controlled studies until now.

Finally, we reviewed reference list to ensure that it is compete and correct.

Thank you.
